# IN UTERO EXPOSURE TO THE DUTCH HUNGER WINTER AND THE PACE OF BIOLOGICAL AGING

**DOI:** 10.1093/geroni/igad104.0680

**Published:** 2023-12-21

**Authors:** Mengling Cheng, Bas Heijmans, Chihua Li, Lambert Lumey, Calen Ryan, Shuang Wang, Jiayi Zhou, Daniel Belsky

**Affiliations:** Swiss Centre of Expertise in Life Course Research, Lausanne, Vaud, Switzerland; Leiden University Medical Center, Leiden, Zuid-Holland, Netherlands; University of Michigan Institute for Social Research, Ann Arbor, Michigan, United States; Columbia University Mailman School of Public Health, New York City, New York, United States; Columbia University Mailman School of Public Health, New York City, New York, United States; Columbia University Mailman School of Public Health, New York City, New York, United States; Columbia University Mailman School of Public Health, New York City, New York, United States; Columbia University, New York City, New York, United States

## Abstract

Adults with histories of early-life adversity experience younger age-of-onset for aging-related disease and shorter lifespan, suggesting an accelerated pace of biological aging. To test this hypothesis, we analyzed data from an established natural experiment: in-utero exposure to famine conditions induced by a Nazi blockade of the Southern Netherlands in Winter 1944-5. We analyzed data from the Dutch Hunger Winter Families Study (DHWFS, N=951). DHWFS enrolled survivors of in-utero famine exposure, matched controls born in the same hospitals as the survivors immediately prior to and following the famine period, and siblings of these individuals. Blood samples were collected when the famine survivors were aged 58. We analyzed blood DNA methylation data to measure the GrimAge and PhenoAge 2nd-generation epigenetic clocks and the DunedinPACE 3rd-generation clock. We tested if famine survivors were biologically older and aging faster as compared to controls. We found that famine survivors had faster DunedinPACE pace of aging, as compared with controls, but similar PhenoAge and GrimAge biological ages. In sex-stratified analyses, we found stronger effects of famine exposure in women as compared with men, including for the GrimAge and PhenoAge clocks; among women, famine exposure was associated with faster DunedinPACE and older GrimAge and PhenoAge, whereas in men effect-sizes were near zero. Famine effects were not accounted for by blood-cell composition and were similar for individuals exposed early and later in gestation. Sibling-difference analyses reproduced size and direction of effect estimates. Findings provide natural-experiment evidence for long-term effects of in-utero famine exposure on the pace biological aging.

